# *ScBx* gene based association analysis of hydroxamate content in rye (*Secale cereale* L.)

**DOI:** 10.1007/s13353-016-0356-3

**Published:** 2016-07-27

**Authors:** Monika Rakoczy-Trojanowska, Wacław Orczyk, Paweł Krajewski, Jan Bocianowski, Anna Stochmal, Mariusz Kowalczyk

**Affiliations:** 1 Warsaw University of Life Sciences, Warsaw, Poland; 2 Centre For Biological Diversity Conservation in Powsin, Polish Academy of Sciences Botanical Garden, Warsaw, Poland; 3 The Plant Breeding And Acclimatization Institute, National Research Institute, Radzików, Poland; 4 Institute of Plant Genetics, Polish Academy of Sciences, Poznań, Poland; 5 Poznań University of Life Sciences, Poznań, Poland; 6 Institute of Soil Science and Plant Cultivation – State Research Institute, Pulawy, Poland

**Keywords:** Candidate gene association mapping, Hydroxamic acids, *ScBx* genes, Single nucleotide polymorphism

## Abstract

Hydroxamates (HX) are major secondary metabolites synthesized by rye and are responsible for some of the unique properties of this cereal, including good tolerance of biotic and abiotic stresses and allelopathy. Recently, five genes encoding enzymes taking part in HX biosynthesis have been sequenced and characterized, which was the starting point to undertake the present study. Association analysis of the content of six HX–HBOA (2-hydroxy-1,4-benzoxazin-3-one), GDIBOA (2,4-dihydroxy-1,4-benzoxazin-3(4H)-one glucoside), DIBOA (2,4-dihydroxy-1,4-benzoxazin-3(4H)-one), GDIMBOA (2,4-dihydroxy-7-methoxy-1,4-benzoxazin-3(4H)-one glucoside), DIMBOA (2,4-dihydroxy-7-methoxy-1,4-benzoxazin-3(4H)-one) and MBOA (6-methoxy-benzoxazolin-2(3H)-one) in the above-ground parts of plants and roots was performed on a population consisting of 102 and 121 diverse inbred lines, in 2013 and 2014, respectively. Altogether, 48 single nucleotide polymorphisms (SNPs) were found to be associated with the content of at least one HX: 20 SNPs were associated with HX synthesized in the above-ground parts of rye plants (AG-SNP), and 28 were associated with HX synthesized in the roots (R-SNP). The highest number of SNPs was present in genes *ScBx1* (9) and *ScBx5* (11). The majority of SNPs were affected by environmental factors, except for two: ScBx4_1702 associated with GDIBOA and MBOA contents, and ScBx5_1105 associated with HBOA content in roots.

**Electronic supplementary material:**

The online version of this article (doi:10.1007/s13353-016-0356-3) contains supplementary material, which is available to authorized users.

## Introduction

HX are secondary metabolites synthesized in numerous species belonging predominantly to the *Poaceae* family, including maize, rye and wheat (Frey et al. 2009, Niemeyer 2009, Makowska et al. 2015). HX have been shown to have many properties typical for secondary metabolites. They play roles in allelopathy and defence against biotic stresses; e.g. European corn borer (ECB, *Ostrinia nubilalis*) in maize (Barry et al. 1994), aphids (*Sitobion avenae*) in wheat (Bohidar et al. 1986) and nematodes in rye (Meyer et al. 2009). HX have also been shown to play a role in improving plant tolerance to soil salinity (Makleit 2005), detoxification of aluminium (Poschenrieder et al. 2005), inhibition of gibberellin-induced α-amylase activity in barley seeds (Kato-Noguchi 2008) and recruitment of plant-beneficial rhizobacteria (Neal et al. 2012). HX are considered to be human health promoting compounds. They lower cancer risk (Roberts et al. 1998) and insulin secretion (Landberg et al. 2010). Moreover, BX have anti-allergic properties (Poupaert et al. 2005) as well as appetite suppression and weight reduction effects (Rosenfeld and Forsberg 2009).

Rye is a species synthesizing HX at spectacularly high levels. Rye HX have been mainly studied in the context of their role in allelopathy (Barnes and Putnam 1987; Tabaglio et al. 2008; Gavazzi et al. 2010) and their toxic impact on nematodes (Zasada et al. 2005, 2007; Meyer et al. 2009). Makleit (2005) reported a positive correlation between DIBOA content in two *Secale* species (*S. cereale* and *S. cereanum*) and their reaction with soil salinity. Because of numerous properties of rye related to HX, effective molecular markers enabling precise selection of forms with an elevated level of these compounds are highly desired. The present study concerns an association analysis based on sequences of five candidate genes: *ScBx1*–*ScBx5*. These genes, recently sequenced and characterized by Bakera et al. (2015), control the first five reactions in HX biosynthesis: from the conversion of indole-3-glycerolphosphate to indole followed by four monooxidations with the final product 2,4-dihydroxy-1,4-benzoxazin-3-one.

The structural polymorphism of *Bx* genes in maize has been successfully used for association mapping by Butrón et al. (2010). The authors identified as many as 182 polymorphisms (71 INDELs and 111 SNPs) in 11 amplicons of genes *Bx1*–*Bx5* and no polymorphisms of greater than 5 % frequency for *Bx8* in a population consisting of 281 diverse inbred lines. Twenty-eight polymorphisms of *Bx1* and one polymorphism of *Bx2* were significantly associated with the content of DIMBOA and DIMBOA-Glc in leaves. Bakera et al. (2015) showed that rye *ScBx* genes present in different rye accessions were generaly similar at the nucleotide level, but a few SNPs and INDELs were found. The highest number of SNPs was identified in the first exon of *ScBx2*, with A/G, C/T and T/C being the most frequent polymorphisms. Of the 19 identified SNPs, three caused non-conserved and one a semi-conserved substitution. Three INDELs were detected in the first exon of *ScBx2* (24-bp deletion), the first exon of *ScBx4* (57-bp insertion) and the second exon of *ScBx5* (3-bp deletion).

The main objective of this study was to identify SNPs in five *ScBx* genes, recently sequenced by a group from the Department of Plant Genetics, Breeding and Biotechnology, Warsaw University of Life Sciences, associated with the content of HX in rye plants by means of the candidate gene association mapping approach.

## Materials and methods

### Plant material

The plant material used in the experiments consisted of a set of diverse inbred lines (DIL) bred in: two Polish breeding companies; the Botanical Garden of Polish Academy of Science, Warsaw University of Life Sciences, Department of Plant Genetics, Breeding and Biotechnology; West Pomeranian University of Technology, Department of Plant Genetics, Breeding and Biotechnology; and Wrocław University of Environmental and Life Sciences, Department of Genetics, Plant Breeding and Seed Production. The lines were selected based on three criteria: good representativeness of the variability of Polish breeding materials, genetic distance determined on their pedigree and minimal impact of inbreeding. Each line was represented by 33 plants. Plants were grown in the experimental field of Warsaw University of Life Sciences (52°08′57.9″N 21°03′51.6″E) in a randomized block design (11 plants per replicate). The experiments were performed in two successive seasons: 2013 and 2014.

### Methods

#### Sample preparation and analysis of HX content

The following HX were analysed: HBOA, GDIBOA, DIBOA, GDIMBOA, DIMBOA and MBOA.

Plant samples—above-ground parts (AG) and roots (R) were collected in the spring when the plants were in the developmental stage GS 20-24 according to the Zadoks Cereal Growth Stage, about two weeks after the start of the vegetation. The sampled tissues were immediately frozen and lyophilized. In 2013, tissue taken from 102 DILs, and in 2014 from 121 DILs were included in the analysis.

Samples of plant material (100 mg d.w.) were mixed with diatomaceous earth, placed in stainless steel extraction cells and extracted with 70 % methanol at 10 MPa operating pressure and 40 °C using an accelerated solvent extraction system (ASE 200, Dionex, Sunnyvale, CA). The cells were filled with LiChroprep RP-18 (40-60 μm, Merck, Germany) in the amount of 250/750 mg for roots and above-ground parts, respectively. After evaporating to dryness under reduced pressure, extracts were reconstituted in 1 ml of methanol containing 0.1 % (v/v) acetic acid and stored at -20 °C. Prior to analyses, extracts were centrifuged for 20 min at 23 000 x g at 4 °C.

Quantitative analyses were carried out on a Waters Acquity UPLC system (Waters, Milford, MA) hyphenated to a triple quadrupole mass spectrometer (Waters TQD). Chromatographic separations were carried out on a Waters BEH C18 column (2.1 × 500 mm, 1.7 μm). The mobile phase A was 0.1 % (v/v) formic acid, and the mobile phase B was acetonitrile containing 0.1 % (v/v) formic acid. A linear gradient from 3 % to 10 % of phase B over 7 min was used to separate HX and their metabolites. The flow rate was 0.7 ml/min and the column was held at 50 °C. Injection volume was 2.5 μl. Each sample was analysed three times. Between injections, the column was washed with 10 volumes of 90 % phase B and then re-equilibrated with 10 volumes of 3 % phase B.

To avoid excessive contamination of the mass spectrometer with polar components of analysed extracts, initial eluate from the column was diverted into waste using a multi-positional valve. After 1.7 min from the start of analysis, the eluate was introduced back into the ion source of the mass spectrometer operating in the negative mode with the following parameters: capillary voltage -2.8 kV, extractor 3 V, RF lens 100 mV, source temperature 130 °C, desolvation temperature 400 °C, desolvation gas flow 1000 l/h, cone gas flow 100 l/h. Collision cell entrance and exit were set to -2 and 0.5, respectively. Parameters of quadrupoles 1 and 3 were optimized to achieve unit-mass resolution. Similarly, cone voltage and collision energy were optimized for each analyte as shown in Table 1.

**Table 1:** Parameters of mass spectrometry analysis

Compound	RT [min]	*m*/*z* of parent [M-H]^-^ ion	*m*/*z* of fragment ion	Collision energy [eV]	Cone voltage [V]
HBOA	2.10	164	108	15	30
DIBOA	2.30	180	134	6	20
GDIBOA	2.80	342	162	15	25
DIMBOA	3.70	210	149	6	15
GDIMBOA	4.60	372	149	15	35
MBOA	5.20	164	149	15	20

Analyses were calibrated from the standard solutions of DIBOA, DIMBOA, HBOA, GDIMBOA and MBOA. GDIMBOA was used as a reference standard for GDIBOA quantitation. Calibration was performed between 2.5 and 30 ng/μl, and it was found to be linear within this range. Samples with higher than 30 ng/μl concentrations of analytes were appropriately diluted (typically between 2 and 5 times) using 0.1 % formic acid and re-analysed. Accuracy of the quantitation was monitored by an injection of quality control sample, consisting of a mixture of all quantitation standards at 8 ng/μl, after each 20 injections of extract samples. Waters MassLynx 4.1 SCN 849 software was used for data acquisition and processing.

#### Genotyping and SNP data processing

In the analysis, the sequences of *ScBx1*÷*ScBx5* genes (Table 2) previously sequenced and characterized in rye inbred line L318 by Bakera et al. (2015) were used. The analysed sequences comprised exons, introns, 3′-UTRs and promoters (except for *ScBx1* in which no 3′-UTRs were assayed). The re-sequencing of *ScBx* genes in the population of DILs was done by a commercial company (Genomed S.A., Warsaw) using the next-generation sequencing (NGS) method. The NGS paired reads (median read length = 190 nt, median number of reads per DIL = 320 587, min = 900, max = 7 211 998) were mapped to the reference sequence using Bowtie 2 (Langmead et al. 2009), which allows for a maximum of 2 mismatches. For processing the mapping results the SNP calling pipeline based on Samtools (mpileup) and Bcftools (index, call) software (Li et al. 2009; Li et al. 2011, software online documentation) were applied. Alleles the same as present in line L318 are named REF and the allele resulting from a given SNP is named ALT. No INDELS were included in the analysis.

**Table 2:** *ScBx* gene sequences included in SNP identification

Gene	Total sequence length including 3′-UTR and promoter [bp]	3′-UTR length [bp]	Promoter length [bp]
*ScBx1* (Acc.No KF636828.1)	4935	317	3000
*ScBx2* (Acc.No KF620524.1 )	2725	75	999
*ScBx3* (Acc.No KF636827.1)	3358	36	1495
*ScBx4* (Acc.No KF636826.1)	3066	166	1041
*ScBx5* (Acc.No KF636825.1 )	3269	0	950

#### Statistical analysis and association mapping

Data were transformed by log_10_(x + 1) and subjected to analysis of variance in the model with fixed effects of years (Y) and random effects of DILs and (DIL x Y) interaction. Fixed-effects analysis of variance was used to test differences between haplotypes obtained by hierarchical clustering of DILs and interaction of these differences with years. Association mapping was done separately for data from two years using the method based on the mixed linear model with population structure estimated by eigenanalysis and modelled by random effects (van Eeuwijk et al. 2010; Malosetti et al. 2013). All analyses were done in Genstat 17 (VSN Int., 2013, https://www.vsni.co.uk).

For promoter analysis: PlantCARE (http://bioinformatics.psb.ugent.be/webtools/plantcare/html/, Lescot et al. 2002).

## Results

### HX content in rye DILs

For all HX in above-ground parts, their content was significantly higher in 2014 than in 2013. The content of DIBOA, DIMBOA and MBOA in roots was higher in 2013. For HBOA and GDIMBOA synthesized in roots, no significant differences between the two seasons were found. Both in 2013 and 2014, the content of HBOA, GDIBOA and DIBOA was higher in AG than in roots, whereas for the remaining HX an opposite relationship was found (Table 3, Fig. SI, Online resource 1).

**Table 3:** Characteristics of the content of HX in AG and R of rye plants

Plant part	HX	Number of DILs		F-statistic for year	Variance components for random effects (std. error)^a)^		Mean (std. error) [μg/g d.w.]	
		2013	2014		Line	Line x Year	2013	2014
AG	HBOA	102	121	28.29***	0.021 (0.010)	0.048 (0.010)	1.01 (0.03)	1.20 (0.02)
	GDIBOA	101	121	322.01***	0.055 (0.014)	0.049 (0.010)	1.65 (0.03)	2.28 (0.03)
	DIBOA	102	120	6.09*	0 (-)	0.030 (0.005)	2.77 (0.02)	2.82 (0.01)
	GDIMBOA	102	121	8.98**	0.042 (0.020)	0.109 (0.021)	0.41 (0.05)	0.57 (0.03)
	DIMBOA	102	121	0.71	0 (-)	0.028 (0.005)	0.05 (0.02)	0.07 (0.01)
	MBOA	102	121	60.99***	0 (-)	0.037 (0.006)	0.01 (0.01)	0.21 (0.02)
R	HBOA	102	120	0.35	0.055 (0.031)	0.183 (0.032)	0.79 (0.07)	0.81 (0.02)
	GDIBOA	102	120	12.13***	0.082 (0.032)	0.184 (0.032)	1.33 (0.06)	1.57 (0.04)
	DIBOA	102	121	109.6***	0.309 (0.069)	0.264 (0.050)	1.80 (0.09)	1.31 (0.06)
	GDIMBOA	102	121	1.89	0.099 (0.020)	0.073 (0.014)	2.49 (0.05)	2.45 (0.04)
	DIMBOA	102	121	14.43***	0.136 (0.055)	0.317 (0.055)	1.46 (0.08)	1.11 (0.05)
	MBOA	102	121	4.29*	0.134 (0.029)	0.105 (0.020)	2.02 (0.06)	1.90 (0.04)

AG – above-ground parts; R – roots

^a)^ Values of 0 put in place of negative estimates of variance components

**Fig. 1:**
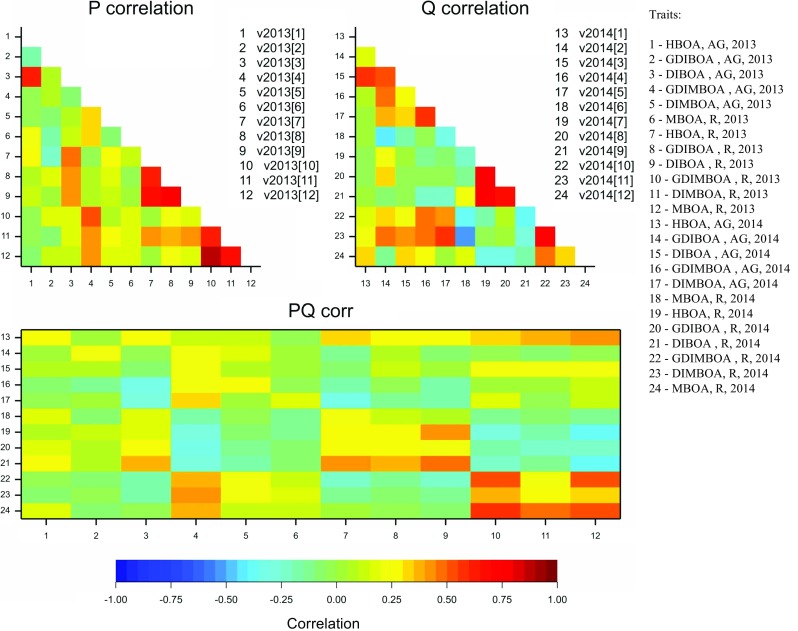
Correlations of HX in above-ground parts (AG) and in roots (R), within years (P – 2013, Q - 2014) and between years (PQ)

A significant positive correlation was observed for the content of two triplets of HX in roots: HBOA, GDIBOA, DIBOA, and GDIMBOA, DIMBOA, MBOA both in a given year and between years (Fig. 1).

### Population structure

Out of the studied DILs, 83 % were homozygous for at least 80 % of SNPs (Fig. SIIa, Online resource 1). Hierarchical clustering of DILs based on SNP data (Manhattan distance based similarity, average link algorithm, grouping threshold 85 %) revealed 11, 12, 10 and 29 haplotypes within genes *ScBx1*, *ScBx2*, *ScBx4* and *ScBx5*, respectively. The haplotypes that occurred in at least 10 DILs in genes *ScBx1*, *ScBx2* and *ScBx4* are described in Table SI, Online resource 1 (no such frequent haplotypes were observed in *ScBx5*). The population structure estimated by eigenanalysis revealed three main subpopulations determined mainly by specific combinations of co-occurring haplotypes of genes *ScBx1* and *ScBx2* (Fig. SIIb, Online resource 1).

### Association analysis

Analysis of variance applied to data concerning HX levels revealed differences significant at P < 0.01 between groups of DILs with specific haplotypes of *ScBx1* and *ScBx2* with respect to the levels of GDIBOA in above-ground parts (p = 0,0001 and 0,003, respectively); in *ScBx1* for DIMBOA in roots (p = 0,006); and in *ScBx4* with respect to the levels of HBOA in above-ground parts (p = 0,009) (Table SII, Online resource 1). Interactions of the differences between haplotypes and year of observation were not significant (P > 0.01).

Altogether, 34 SNPs were found to be associated with the content of, at least, one HX: 20 SNPs were associated with HX synthesized in the above-ground parts of rye plants (AG-SNP) and 28 in the roots (R-SNP). Allele substitution effects were higher for roots than for leaves (Tables SIII and SIV – Online resource 1). Fourteen SNPs — ScBx1_2598, ScBx1_4891, ScBx2_140, ScBx2_180, ScBx2_514, ScBx2_526, ScBx5_70, ScBx5_219, ScBx5_270, ScBx5_359, ScBx5_621, ScBx5_661, ScBx5_755, ScBx5_1105 — were common for HX in the above-ground parts and roots (Tables SIII and SIV – Online resource 1) and 20 SNPs — ScBx1_198, ScBx1_650, ScBx1_1646, ScBx1_2668, ScBx1_3853, ScBx1_4219, ScBx1_4515, ScBx2_571, ScBx2_1780, ScBx3_2137, ScBx4_511, ScBx4_1583, ScBx4_1607, ScBx4_1627, ScBx4_1650, ScBx4_1702, ScBx4_1703, ScBx5_698, ScBx5_705, ScBx5_782 — were unique either for HX content in above-ground parts or in rots. Among common SNPs, seven SNPs identified in the *ScBx5* gene — ScBx5_70, ScBx5_219, ScBx5_270, ScBx5_359, ScBx5_661, ScBx5_755 and ScBx5_1105 — were associated with the content of at least one (the same) HX, mostly with DIMBOA (Table 4). Among a set of 20 (six AG-SNP, 14 R-SNP) unique markers, an association with the content of more than one HX was found for 12 markers (five AG-SNP, seven R-SNP) (Tables SIII and SIV — Online resource 1). The number of SNPs detected in *ScBx* genes varied and was: 9 for *ScBx1* (including two SNPs common for HX content in above-ground parts and roots), six for *ScBx2* (four common SNPs), one for *ScBx3* (only for HX content in above-ground parts), seven for *ScBx4* (no common SNPs) and 11 for *ScBx5* (eight common SNPs). Nearly 60 % of SNPs were present in the gene promoter sequences (Tables SIII and SIV – Online resource 1).

**Table 4:** Markers common for the content of HX in above-ground parts (AG) and roots (R) associated with the content of one or more HX

Marker ID	Plant part	Association with HX	2013		2014	
			S	E	S	E
ScBx5_70	AG	DIMBOA			3.51	-0.07
	R				1.42	-0.15
ScBx5_219	AG	DIMBOA			3.74	-0.07
	R				2.06	-0.18
ScBx5_270	AG	DIMBOA	2.92	0.08		
	R		3.15	0.38		
ScBx5_359	AG	DIMBOA			3.43	-0.07
	R				1.83	-0.17
ScBx5_661	AG	GDIBOA	1.93	0.09		
	R		1.30	0.16		
ScBx5_755	AG	DIMBOA	1.68	0.06		
	R		1.70	0.26		
ScBx5_1105	AG	DIMBOA			1.38	-0.05
	R				1.33	-0.15

S – -log10(P-value) statistics

E – allelic effect

The majority (82.4 %) of SNPs were found in non-coding sequences (21 SNPs in promoters, six in introns and one in 3’UTR) and only six SNPs in coding sequences od *ScBx* genes.

All AG-SNP effects were affected by the environmental factors, whereas for two R-SNPs — ScBx4_1702 associated with GDIBOA and MBOA contents, and ScBx5_1105 associated with HBOA content — the effects were environmentally independent. Both SNPs with environmentally independent effects were localized in the coding sequences — in the second exon of *ScBx4* and in the first exon of *ScBx5* (Table 5, Tables SIII, SIV, SV – Online resource 1). The SNP in *ScBx4* gene results in methionine/valine substitution whereas the second one SNP has no effect on amino acid level.

**Table 5:** Impact of SNPs in *ScBx* gene coding sequences on amino acid substitution

Marker ID	SNP	SNP position	Codone change	AA substitution
ScBx1_4515	T/C	7^th^ Ex	TGG/CGG	Y/R
ScBx2_1780	A/G	1^st^ Ex	AAG/AGG	K/R
ScBx3_2137	C/T	2^nd^ Ex	TGC/TGT	-
**ScBx4**_**1702**	A/G	2^nd^ Ex	ATG/GTG ATG/GCG ATG/ACG	M/V M/A M/T
ScBx4_1703	T/C			
**ScBx5**_**1105**	G/T	1^st^ Ex	CCG/CCT	-

Bold fonts – markers associated with HX content independently of an environment

A summary of the association analysis is presented in Table 6.

**Table 6:** Summary of the association analysis of HX content in rye plants

No. of SNPs significantly associated with HX content/ markers common for HX content in AG and R (independent of environment)	No. of SNPs significantly associated with HX content in each *ScBx* gene	No. of SNPs significantly associated with HX content in AG/ unique markers (independent of environment)	No. of SNPs significantly associated with HX content in R/ unique markers (independent of environment)	Markers common for HX content in AG and R (independent of environment)	Markers associated with the content of more than one HX (independent of environment)
48/20 (2)	*ScBx1* - 9 *ScBx2* - 6 *ScBx3* - 1 *ScBx4* - 7 *ScBx5* - 11	20/6 (0)	28/14 (2)	14 (1)	12 (1)

AG – above-ground parts

R – roots

## Discussion

Taking into consideration the role of HX in rye and having at our disposal full sequences of five *ScBx* genes controlling their biosynthesis, we decided to perform candidate gene association mapping to develop molecular markers for the selection of forms with enhanced HX content. For this, five genes, *ScBx1*÷*ScBx5*, with a proven role in the biosynthesis of HX were selected.

Association mapping involves searching for genotype (usually individual SNPs or SNP haplotypes)–phenotype correlations in unrelated individuals using dedicated statistical methods (Abdurakhmonov and Abdukarimov 2008; Zhu et al. 2008; Rafalski 2010) and makes it possible to generate good quality markers. There are two mapping approaches: genome wide association mapping (GWAM) and candidate gene association mapping (CGAM). The latter is a powerful tool providing good recognition of genes controlling traits of interest. To date, CGAM has been successfully used in rye for identifying markers for frost tolerance (FT) (Li et al. 2011). The authors found 120 statistically significant SNPs in 12 candidate genes, selected for analysis due to their putative role in the FT, chosen from previous studies employing bi-parental linkage mapping and expression analysis. The number of SNPs significantly associated with FT depended on experimental design with the highest number—69 SNPs in nine genes (*ScCbf2*, *ScCbf9b*, *ScCbf11*, *ScCbf12*, *ScCbf15*, *ScDhn1*, *ScDhn3*, *ScDreb2*, *ScIce2*) in the controlled platform. Thirty-three SNPs from six genes were significantly associated with FT in at least two of the three platforms.

In both seasons, the content of HBOA, GDIBOA and DIBOA in above-ground parts was significantly higher than in roots. It is well known that in rye DIBOA and GDIBOA are predominant in aerial parts, whereas DIMBOA and MBOA are predominant in roots (Frey et al. 2009; Zasada et al. 2007; Meyer et al. 2009). For DIBOA and GDIBOA, compounds dominant in above-ground parts, our results are consistent with the previous study performed in the Institute of Soil Science and Plant Cultivation which reported their content in the aerial parts to be, respectively, five and two times higher than in the roots (Krzyżanowska et al. 2006). However, the differences between above-ground parts and roots were much higher than in our research; for both compounds we noted two times higher amounts of them in above-ground parts than in the roots. It seems logical that content of HBOA in above-ground parts of rye plants should also be higher than in roots since the DIBOA arises directly from this HX.

In the case of roots, the dominant compounds were GDIMBOA and MBOA, but of the six examined HX only GDIBOA was synthesized at a higher level in the second year of experiments. The reasons for the different relationship between above-ground parts of plants and roots with respect to the content of at least three (DIBOA, DIMBOA and MBOA) out of six HX may be the completely different springtime weather in successive years in which tissues were sampled (Table SVI, Online resource 1). As reviewed by Niemeyer (2009), the profile and dynamics of HX biosynthesis are strongly affected by environmental conditions. The warm, humid weather with high rainfall in spring in 2014 must have had a negative impact on the synthesis of DIBOA, DIMBOA and MBOA in roots.

Despite such substantial differences of atmospheric conditions, a significant positive correlation was observed for the content of two triplets of HX in roots: HBOA, GDIBOA, DIBOA, and GDIMBOA, DIMBOA, MBOA, both in a given year and between years. It looks obvious as the first triplet consists of products of three consecutive reactions controlled by genes *ScBx5*, *gt* and *glu*. Two (DIMBOA, GDIMBOA) out of three compounds of the second triplet are synthesized in two successive reactions driven by the *glu* gene. GDIMBOA is further methylated into HDMBOA-Glc and, subsequently, under the action of β-glucosidases on HDMBOA-Glc, HDMBOA is released and is degraded quickly to MBOA (Oikawa et al. 2002). The correlation between MBOA and the rest of HX content in the roots does not agree with the result of previous research reported by Carlsen et al. (2009), who found no correlation between MBOA and all other benzoxazinoids in the roots of rye and concluded that it might indicate that a hitherto unknown synthetic pathway exists for MBOA. Moreover, the markers identified in our work, ScBx4_1627, ScBx4_1650, ScBx4_1702, ScBx4_1703, ScBx5_270, ScBx5_705 (among them one marker, ScBx4_1702, is independent of the environment), common for the content of MBOA and other HX in roots (Table SIV, Online resource 1), contradicts the thesis formulated by Carlsen et al. (2009) and supports the existence of a strong correlation between the root content of MBOA and at least GDIMBOA and DIMBOA.

The haplotypes of genes *ScBx1* and *ScBx2* formed three combined haplotypes occurring together and explaining to a large extent the division of the studied population into subpopulations. Nevertheless, this population structure and haplotypes of other genes did not explain fully the differences in HX levels observed in above- and below-ground parts of plants. The association analysis performed for individual SNPs resulted in finding polymorphisms affecting the HX levels. In the case of above-ground parts, all SNPs in *ScBx1*, *ScBx2* and *ScBx4* found to be associated with HX exhibited substantial polymorphisms within haplotype groups; for root HX, this was true for most of the significant SNPs. This suggests the importance of single nucleotide polymorphisms for the variation in HX levels.

Altogether, 34 SNP markers significantly associated with the content of one or, in most cases, more than one HX were detected. The majority of them were found in the non-coding sequences—promoters and introns, and one SNP—in 3’UTR. The structural changes in promoters can influence transcription efficiency and, therefore, HX production. A significant positive correlation between the expression level of *ScBx1* gene and the content of HX in rye plants has been shown by Groszyk et al. (2013, 2015). La Hovary (2012) found that BX1 and BX2 transcript level paralleled the HX content. In case of SNPs present in introns, the modification of protein composition and properties resulted from an alternative splicing cannot be excluded. Six SNPs were identified in the coding sequences of ScBx genes. They usually caused the AA substitution, and, probably, the activity of encoded enzymes. Out of two environmentally stable polymorphisms ScBx5_1105 (G/T in the first exon) did not affect the amino acid substitution. The second stable SNP ScBx4_1702 (A/G in the second exon) does change methionine to valine. Moreover, the adjacent ScBx4_1703 (T/C) also causes AA substitution (M/T) and the possible combination of these two SNPs may cause three possible substitutions: M/V, M/T, M/A. However, as only ScBx4_1702 was proved to be associated with HX content in roots—the substitution M/V should be considered as a reason for differences in HX content.

Allele substitution effects were usually much higher for roots than for the above ground parts of plants which can be caused by different, organ-specific regulation of *ScBx* gene expression.

The number of identified SNPs differed between individual genes. The highest number of SNPs was detected in genes *ScBx1* and *ScBx5* (nine and 11, respectively, including those common for HX content in the above-ground parts and roots). In the study of Butrón et al. (2010), which is the only point of reference (according to our best knowledge), the polymorphisms significantly associated with DIMBOA and GDIMBOA content in maize whorl tissue centred at the highest ligule sampled 38 days after planting were detected exclusively across the four amplicons in *ZmBx1* and in one amplicon for the *ZmBx2* gene. Surprisingly, although candidate association analysis determined that the sequence polymorphisms at *ZmBx1* greatly affected variation of DIMBOA content in a diverse panel of maize inbreds, no specific causal polymorphism or polymorphisms responsible for the quantitative trait loci (QTL) detected using the linkage mapping approach were identified.

Out of 34 markers (including 14 associated with the content of HX both in the upper parts of plants and roots and seven with more than one HX), only some of them may have practical significance. Unfortunately, regardless of a strong, significant association, the majority of SNPs were influenced by the environment (unlike between-haplotype differences, which were more stable). Moreover, some markers, namely SNPs in the *ScBx5* gene (ScBx5_70, ScBx5_219, ScBx5_270 and ScBx5_359), seem to be controversial despite their proven significant association with HBOA content, both in the above-ground parts of plants and roots. It is difficult to find a relationship between the structural variation of the *ScBx5* gene and HBOA content as *ScBx5* controls the transformation of HBOA into DIBOA. So, all HX preceding DIBOA should not be associated with the structural variation of the *ScBx5* gene. However, hitherto unknown feedback between DIBOA and HBOA cannot be excluded. In maize, DIMBOA repressed *Bx* gene expression in a dose-dependent manner, acting as a signal for transcriptional feedback of BX biosynthesis (Ahmad et al. 2011).

Another drawback was the relatively high proportion of “zero” (below detection level) measurements in the case of DIMBOA and MBOA in 2013. Therefore, conclusions about associations with DIMBOA and MBOA should be cautious.

The most valuable SNPs identified in the present study are two: R-SNPs: ScBx5_1105 associated with HBOA content and ScBx4_1702 associated with GDIBOA and MBOA contents and also with PHS-R. Both markers were shown to be environmentally independent. As pointed out above, the weather conditions were drastically different in the two experimental seasons. Thus, the markers were subjected to severe selection pressure which additionally validates their value and universality. Moreover, using phenotyping data from a parallel experiment, it was found that one of them, ScBx2_1702, was stably associated with pre-harvest sprouting, considered to be a trait potentially dependent on HX, in two successive years. However, the relationship between the content of HX in roots and the trait expressed in grains remains unclear.

Two SNP (ScBx5_1105 and ScBx4_1702)-based markers associated with the content of protective HX in roots can be recommended as a useful molecular tool for selection of rye forms improved in terms of HX dependent properties, i.e. with improved adaptability to soil salinity, tolerance to soil nematodes and allelopathic impacts.

## Electronic supplementary material

Below is the link to the electronic supplementary material.

ESM 1 (PDF 539 kb)
