# Supplementary material for: ScBx gene based association analysis of hydroxamate content in rye (Secale cereale L.)
Source: J Appl Genet. 2016 Jul 27;58(1):1–9. doi: 10.1007/s13353-016-0356-3 (PMC5243912; doi:10.1007/s13353-016-0356-3)
Supplement: Supplementary file 1 — (PDF 539 kb) [file 13353_2016_356_MOESM1_ESM.pdf]

ScBx gene based association analysis of hydroxamate content in rye (_Secale cereale_ L.)

JOURNAL OF APPLIED GENETICS

Monika Rakoczy-Trojanowska1,2, Wacław Orczyk3, Paweł Krajewski4, Jan Bocianowski5, Anna Stochmal6, Mariusz Kowalczyk6
1)Warsaw University of Life Sciences, Warsaw, Poland, 2)Polish Academy of Sciences Botanical Garden – Centre For Biological Diversity Conservation in Powsin Warsaw, Poland, 3)The Plant Breeding And Acclimatization Institute – National Research Institute, Radzików, Poland 4)Institute of Plant Genetics, Polish Academy of Sciences, Poznań, Poland, 5)Poznań University of Life Sciences, Poznań, Poland, 6) Institute of Soil Science and Plant Cultivation – State Research Institute, Pulawy

e-mail address of the corresponding author: monika_rakoczy_trojanowska@sggw.pl

Table SI. Haplotypes of genes *ScBx1*, *ScBx2* and *ScBx4* most frequent in the studied population of DILs.

Notation: A, T, G, C - nucleotides; two nucleotides separated by comma - polymorphism within haplotype group; nucleotide in brackets - nucleotide with minor frequency in the group (less than 10%)

SNP in gene *ScBx1*	Haplotype		
	1	2	3
ScBx1_198	T	C	C
ScBx1_390	G	T(G)	G
ScBx1_650	T(C)	T	T
ScBx1_714	G	A(G)	G
ScBx1_1233	G	A(G)	G
ScBx1_1367	A(G)	G	G
ScBx1_1646	T,C	T,C	T,C
ScBx1_1673	T(C)	C	C
ScBx1_1766	T	C	T
ScBx1_2006	C	A	C
ScBx1_2072	A	G(A)	A
ScBx1_2308	T	G	T
ScBx1_2474	A(C)	C	C
ScBx1_2598	T	G(T)	G,T
ScBx1_2668	T(C)	C(T)	C
ScBx1_2717	T	C(T)	T
ScBx1_3853	C(T)	T	T
ScBx1_3976	G	A(G)	G
ScBx1_4219	C,G	G	C,G
ScBx1_4453	G	A	G
ScBx1_4491	A	G(A)	A
ScBx1_4515	C	T	T
ScBx1_4663	G(A)	A	A
ScBx1_4736	C(G)	G	G
ScBx1_4794	T	C(T)	T
ScBx1_4891	G	T(G)	G,T
Number of DILs	29	51	41

SNP in gene *ScBx2*	Haplotype		
	1	2	3
ScBx2_46	A	G	G
ScBx2_79	T	C	T
ScBx2_140	T	G	G,T
ScBx2_180	T	C	C,T
ScBx2_212	A	G	G
ScBx2_417	A	G(A)	A
ScBx2_506	C	A	C
ScBx2_514	A(G)	G	A,G
ScBx2_526	A	A,C	A
ScBx2_566	G	A	G
ScBx2_571	C	C,T	C
ScBx2_1345	T	C	T
ScBx2_1457	G(A)	A	A
ScBx2_1458	C(A)	A	A
ScBx2_1462	T(C)	C	C
ScBx2_1525	C	T	C
ScBx2_1780	A	A,G	G
ScBx2_1782	C	T	C
ScBx2_1810	T(C)	C	C
ScBx2_1913	T(C)	C	C
ScBx2_2213	C	T(C)	C
ScBx2_2614	A(G)	G	G
Number od DILs	45	45	30

SNP in gene *ScBx4*	Haplotype			
	1	2	3	4
ScBx4_511	A	G	A(G)	G(A)
ScBx4_1583	T	C	C,T	T
ScBx4_1607	G,T	T(G)	T(G)	G
ScBx4_1627	C	C	C	T
ScBx4_1650	C	C	C	T
ScBx4_1702	G	G	G	A
ScBx4_1703	C	C	C	T
Number od DILs	64	17	13	24

Table SII. Results of testing differences between haplotypes with respect to HX content

Plant part	HX	Mean for haplotype in *ScBx1*				P-value[a)]	Mean for haplotype in *ScBx2*				P-value[a)]	Mean for haplotype in *ScBx4*					P-value[a)]
		1	2	3	Other		1	2	3	Other		1	2	3	4	Other	
AG	HBOA	1.14	1.13	1.06	1.14	0.339	1.12	1.12	1.06	1.20	0.101	1.11	1.14	0.93	1.17	1.17	0.009**
	GDIBOA	1.93	2.03	1.89	2.14	0.001**	2.02	1.95	1.90	2.13	0.003**	1.94	1.93	2.14	1.99	2.05	0.047*
	DIBOA	2.81	2.80	2.77	2.81	0.641	2.80	2.80	2.78	2.82	0.607	2.80	2.79	2.73	2.84	2.81	0.200
	GDIMBOA	0.47	0.52	0.49	0.47	0.878	0.53	0.45	0.46	0.53	0.625	0.50	0.41	0.58	0.39	0.55	0.350
	DIMBOA	0.06	0.09	0.06	0.01	0.193	0.08	0.05	0.05	0.05	0.719	0.06	0.02	0.06	0.04	0.09	0.573
	MBOA	0.14	0.12	0.13	0.08	0.381	0.11	0.13	0.14	0.10	0.639	0.15	0.08	0.04	0.10	0.12	0.089
R	HBOA	0.83	0.76	0.90	0.72	0.263	0.73	0.80	0.89	0.80	0.327	0.83	0.71	0.73	1.01	0.70	0.063
	GDIBOA	1.41	1.39	1.56	1.52	0.263	1.36	1.37	1.55	1.59	0.056	1.39	1.33	1.54	1.66	1.51	0.097
	DIBOA	1.61	1.40	1.75	1.39	0.035*	1.36	1.58	1.73	1.47	0.051	1.54	1.42	1.48	1.92	1.34	0.036*
	GDIMBOA	2.40	2.53	2.48	2.40	0.271	2.51	2.42	2.49	2.42	0.617	2.51	2.44	2.62	2.30	2.41	0.060
	DIMBOA	1.19	1.37	1.40	0.95	0.006**	1.35	1.19	1.39	1.03	0.043*	1.39	1.08	1.50	1.14	1.08	0.021*
	MBOA	1.89	2.00	2.03	1.84	0.214	1.98	1.89	2.05	1.86	0.189	2.02	2.08	2.02	1.75	1.87	0.052

a) P-value from ANOVA performed to test differences among groups of lines with haplotypes defined in Table SI and an additional group consisting of all other haplotypes.

*,** - differences significant at $P < 0.05$, 0.01, respectively

Table SIII. Associations between SNP and HX content in above-ground parts of plants, in CGAM at P 0,05 with allelic substitutions effects */**)

SNP ID	SNP position	Allele**/***)		Frequency of allele		HBOA				GDIBOA				DIBOA				GDIMBOA				DIMBOA				MBOA			
						2013		2014		2013		2014		2013		2014		2013		2014		2013		2014		2013		2014	
		R	A	R	A	S	E	S	E	S	E	S	E	S	E	S	E	S	E	S	E	S	E	S	E	S	E	S	E
ScBx1_1646	P	T	C	0.78	0.22																			2.03	-0.07	2.04	0.03		
ScBx1_2598	P	G	T	0.41	0.59							2.02	0.17																
ScBx1_4891	3'UTR	T	G	0.43	0.57							3.51	0.24	1.57	-0.09														
ScBx2_140	P	G	T	0.47	0.53			1.36	-0.09			1.47	0.12	2.55	-0.11														
ScBx2_180	P	C	T	0.47	0.53			1.36	-0.09			1.45	0.12	2.55	-0.11														
ScBx2_514	P	G	A	0.50	0.50			1.59	-0.10					3.03	-0.12									1.88	0.08				
ScBx2_526	P	C	A	0.21	0.79													1.35	-0.17							2.54	-0.03	4.22	0.14
ScBx2_571	P	T	C	0.21	0.79													1.35	-0.17							2.54	-0.03	3.95	0.14
ScBx2_1780	1st Ex	A	G	0.55	0.45													1.41	-0.12							2.22	-0.02	2.07	0.07
ScBx3_2137	2nd Ex	C	T	0.52	0.45																							1.38	0.04
ScBx4_1583	1st I	T	C	0.79	0.21	1.72	-0.10			2.18	0.09																	2.15	-0.08
ScBx4_1607	1st I	G	T	0.77	0.23					1.38	0.10									1.63	-0.15	1.32	-0.09						
ScBx5_70	P	T	G	0.41	0.59			1.98	0.07															3.51	-0.07				
ScBx5_219	P	C	T	0.38	0.62			1.64	0.06															3.74	-0.07				
ScBx5_270	P	A	T	0.72	0.28			1.31	0.06			1.69	-0.10									2.92	0.08	1.99	-0.06				
ScBx5_359	P	G	A	0.37	0.63			1.37	0.06															3.43	-0.07				
ScBx5_621	P	T	C	0.78	0.22					1.38	0.08																		
ScBx5_661	P	G	C	0.77	0.23					1.93	0.09																		
ScBx5_755	P	A	T	0.74	0.26							3.57	-0.15									1.68	0.06	1.48	-0.05				
ScBx5_1105	1st Ex	G	T	0.23	0.77							1.34	-0.08	1.87	0.07					1.64	-0.11			1.38	-0.05				

R – allele present in the reference line L318

A – allele with SNP

P - promoter

Ex - exon

I - intron

S - -log10(P-value) statistics

E – allele effect

*/**) Effect of allele A with respect to allele R

Table SIV. Associations between SNP and HX content in roots, in CGAM at P 0,05 with allelic substitutions effects */**)

SNP ID	SNP position	Allele		Freq. of allele		HBOA				GDIBOA				DIBOA				GDIMBOA				DIMBOA				MBOA			
						2013		2014		2013		2014		2013		2014		2013		2014		2013		2014		2013		2014	
		R	A	R	A	S	E	S	E	S	E	S	E	S	E	S	E	S	E	S	E	S	E	S	E	S	E	S	E
ScBx1_198	P	C	T	0.57	0.43													1.37	0.18			1.53	0.33						
ScBx1_650	P	T	C	0.58	0.42																	1.37	0.31						
ScBx1_2598	P	G	T	0.41	0.59	1.72	-0.33																			1.45	0.27		
ScBx1_2668	P	C	T	0.58	0.42																	1.51	0.33						
ScBx1_3853	3th I	T	C	0.60	0.40																	1.68	0.35						
ScBx1_4219	5th I	G	C	0.69	0.31																					1.42	-0.24		
ScBx1_4515	7th Ex	T	C	0.57	0.43																	1.57	0.33						
ScBx1_4891	3'UTR	T	G	0.43	0.57	1.74	-0.34													1.59	0.20								
ScBx2_140	P	G	T	0.47	0.53	1.68	-0.31																						
ScBx2_180	P	C	T	0.47	0.53	1.68	-0.31																						
ScBx2_514	P	G	A	0.50	0.50	1.75	-0.31																						
ScBx2_526	P	C	A	0.21	0.79			1.34	0.08																				
ScBx4_511	P	G	A	0.40	0.60													1.65	0.12					2.55	0.17				
ScBx4_1627	1st I	T	C	0.21	0.79	1.93	-0.24			1.31	-0.18					1.69	-0.20	1.54	0.14					1.51	0.16	1.63	0.19		
ScBx4_1650	1st I	T	C	0.23	0.77	1.93	-0.24			1.31	-0.18					1.48	-0.17	1.54	0.14					1.35	0.14	1.63	0.19		
**ScBx4_1702**	2nd Ex	A	G	0.22	0.78	1.93	-0.24			**1.31**	**-0.18**	**1.39**	**-0.10**			1.65	-0.19	1.54	0.14							**1.63**	**0.19**	**1.45**	**0.10**
ScBx4_1703	2nd Ex	T	C	0.21	0.79	1.93	-0.24			1.31	-0.18					1.76	-0.21	1.54	0.14					1.36	0.15	1.63	0.19		
ScBx5_70	P	T	G	0.41	0.59	2.06	0.21																	1.42	-0.15				
ScBx5_219	P	C	T	0.38	0.62	1.96	0.20																	2.06	-0.18				
ScBx5_270	P	A	T	0.72	0.28	1.53	0.21															3.15	0.38			1.45	0.19		
ScBx5_359	P	G	A	0.37	0.63	2.62	0.24							1.55	0.22							1.30	0.19	1.83	-0.17				
ScBx5_621	P	T	C	0.78	0.22																	2.21	0.30						
ScBx5_661	P	G	C	0.77	0.23					1.30	0.16			1.34	0.22							2.32	0.30						
ScBx5_698	P	T	C	0.63	0.37																	1.60	0.22						
ScBx5_705	P	C	T	0.63	0.37																	1.59	0.22			1.38	0.15		
ScBx5_755	P	A	T	0.74	0.26	1.32	0.19															1.70	0.26						
ScBx5_782	P	G	T	0.63	0.37																	1.86	0.24						
**ScBx5_1105**	1st Ex	G	T	0.23	0.77	**1.49**	**0.21**	**1.98**	**0.09**							2.18	0.24							1.33	-0.15				

R – allele present in the reference line L318

A – allele with SNP

P - promoter

Ex - exon

I - intron

S - -log10(P-value) statistics

E – allele effect

*/**) Effect of allele A with respect to allele R

Bold fonts – markers associated with a given HX content independently of an environment

Table SV. Characteristics of the weather in the spring of 2013 and 2014 in Poland

Paratmeter	Season	
	2013	2014
Temperature [^{0}C]	day – 10.9<br>night – 6.3	day – 13.3<br>night – 10.5
Rainfall [mm]	2.6	9.8
Relative humidity [%]	67.2	69.8

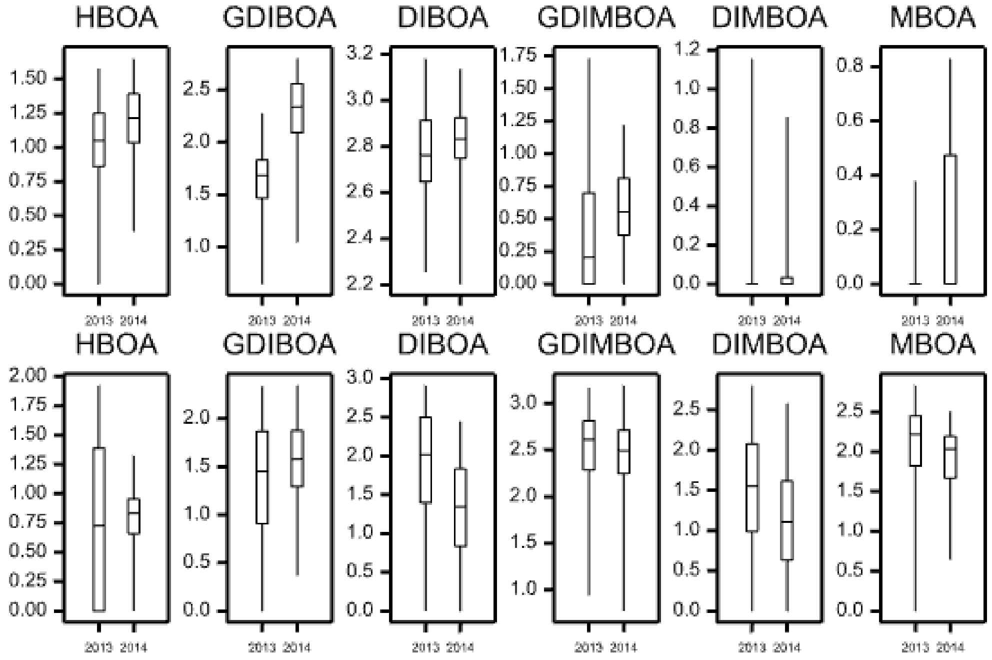

Fig. SI. Variability of DILs with respect to HX content in above ground parts of palns (upper panel) and roots (lower panel) in two years of observations (log scale)
